# Physician-modified “fenestrated” Gore thoracic branch endoprosthesis (TBE) for zone 0 hybrid arch repair

**DOI:** 10.1016/j.jvscit.2026.102130

**Published:** 2026-01-13

**Authors:** George Pontikis, Cheong Jun Lee, Gregory Estrera, Fernando Fleischman, Sukgu M. Han

**Affiliations:** a Duly Health and Care, Naperville, IL; b Endeavor Health, Evanston, IL; c University of Southern California, Los Angeles, CA

**Keywords:** Fenestrated endovascular aortic arch repair, Fen TBE

## Abstract

Despite advances in endovascular repair of aortic arch pathologies, the gold standard remains open surgical repair. Two single-branched options for zone 0 exist in the United States for high-risk patients. Both systems require adjunctive multiple cervical debranching, which carries significant morbidity. We describe a practical endovascular solution that simplifies modification, implantation, and adjunctive cervical debranching, by performing a single left carotid transposition and creating an additional fenestration within the existing Gore thoracic branch endoprosthesis platform. We present our initial series of six patients.

Despite advances in endovascular repair of aortic arch pathologies, the gold standard remains open surgical repair.^1^, ^2^, ^3^, ^4^ Arch branched and fenestrated endograft systems offer promise for hybrid or total endovascular treatment of aortic arch pathologies.^5^, ^6^, ^7^, ^8^, ^9^ Two single-branched options for zone 0 exist in the United States for patients who are high-risk for open repair: the Gore thoracic branch endoprosthesis (TBE) (W. L. Gore & Associates), which recently obtained United States Food and Drug Administration commercial approval for zone 0 indication, and the NEXUS Arch Stent Graft System (Endospan Ltd), which is actively enrolling in a pivotal trial.^7^^,^^8^ Both device systems require adjunctive multiple cervical debranching procedures, which carry their own associated risks. Alternative treatment options include physician-modified fenestrated branched endovascular grafts or in situ fenestration.^10^ However, the high procedural complexity, as well as the poorly defined risks of severe complications, have limited the adoption of these procedures to a small number of high-volume centers. We describe a practical endovascular solution that simplifies modification and implantation, as well as adjunctive cervical debranching, by creating an additional fenestration within the existing Gore TBE platform (FEN-TBE). In addition to the technique, we present our initial series of six patients and their outcomes. This is an off-instructions for use (IFU) modification of the Gore TBE system that is not endorsed or approved by the manufacturer or the United States Food and Drug Administration. As part of the informed consent process, we discuss all on-label and off-label treatment options, as well as those involving investigational procedures. We also discuss the risks associated with each option, including available published data and our center outcomes. Consent was obtained from the patients for the procedure and use of images and data for this publication.

## Methods

### Modification

**Fig 1:**
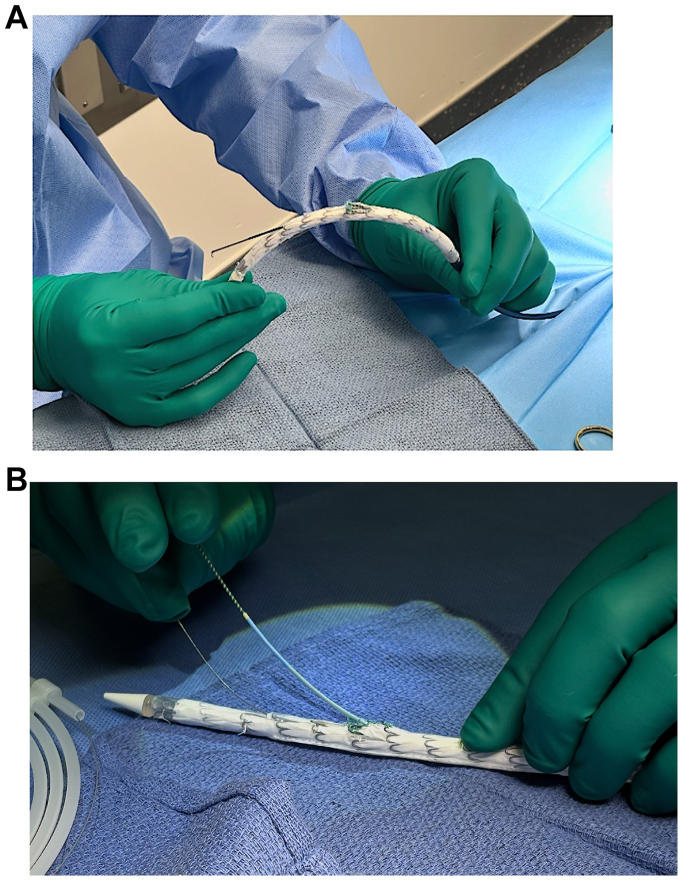
**(A)** An additional fenestration within the existing Gore thoracic branch endoprosthesis platform (FEN-TBE) graft with single reinforced fenestration. **(B)** An 018 wire is placed through the removable guide tube into the portal and preloaded 4Fr JR catheter with 035 wire is placed through the fenestration.

A Gore TBE device is sized based on the diameter of the proximal seal zone. We have used both the 15-cm and 20-cm length TBEs with 12-mm portal. The site of the fenestration is selected based on the innominate to left subclavian artery (LSA) distance along the outer curve of the device. The minimum distance should be at least 25 mm from the distal edge of the innominate to the center of the LSA. This avoids shuttering of the portal branch by the fenestrated branch. With a #11 blade, the deployment sleeves are opened to expose the stent graft fabric. This is extended with Potts scissors with care to avoid the deployment line on the inner curve. A 10-mm fenestration is cut down using a combination of knife and scissors without the use of thermal cautery. The fenestration is then reinforced with a 10-mm Goose neck snare or 018 nitinol micropuncture access wire with a running-locking 4-0 Ethibond or CV6 Goretex sutures. The TBE removable guide tube is used to preload an 0.018” wire, then the lumen of the TBE device is accessed through the fenestration with an 035-dilator directed to the bottom of the device. A stiff wire is introduced through the dilator and used to exchange for a 4Fr JR4 catheter that is introduced from the bottom of the device out the fenestration. This creates a double vessel FEN-TBE, containing a pre-wired innominate portal, and a pre-catheterized LSA fenestration. The device is then gently pre-curved prior to introduction into the patient to assist with device tracking and orientation (Fig 1). It is important to note that there is limited space within the device to accommodate the preloaded 4Fr JR4 catheter for the fenestration. Thus an 0.018 wire is used for the through and through wire to accommodate the preloaded catheter. This allows for less internal crowding and more maneuverability of the preloaded catheter for cannulation within the undeployed device.

### Implantation

**Fig 2:**
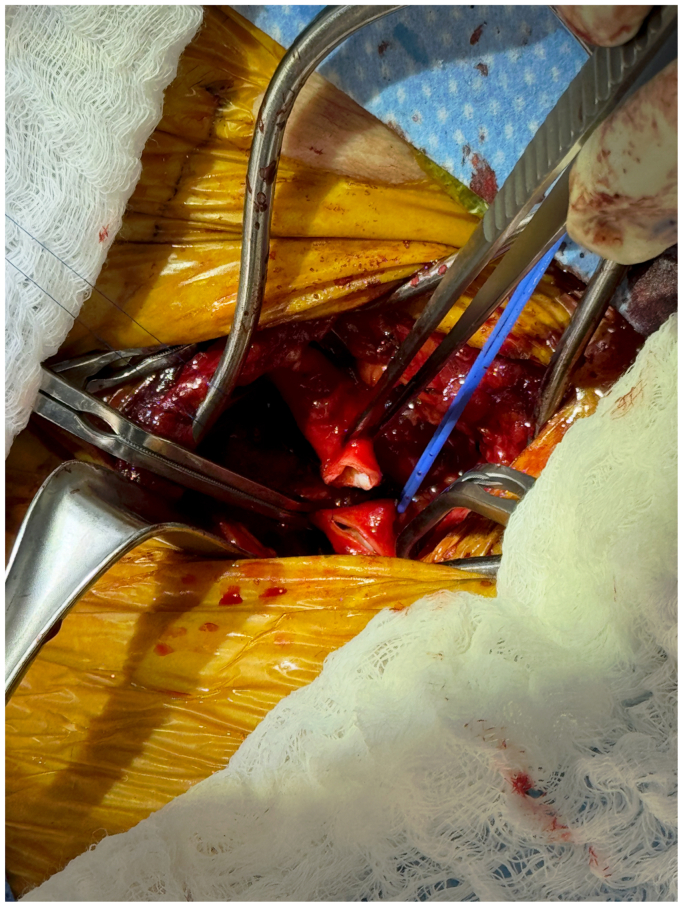
Left carotid transposition on to left subclavian artery (LSA).

The left common carotid artery is transposed to the LSA prior to the zone 0 thoracic endovascular aortic repair (Fig 2). Right radial access is obtained to facilitate the innominate branch component placement. The initial setup with the right radial to femoral through-and-through access is performed in accordance with the IFU. A catheter is exteriorized over the through-and-through wire to allow passage of the preloaded 0.018” wire.

Prior to insertion of the FEN-TBE device, heparinized saline is flushed through the fenestration by use of a 5Fr dilator as means to displace the air from the center of the device. We also allow back bleeding with insertion of the device into the sheath for 15 to 20 seconds to further decrease the risk of embolism.

**Fig 3:**
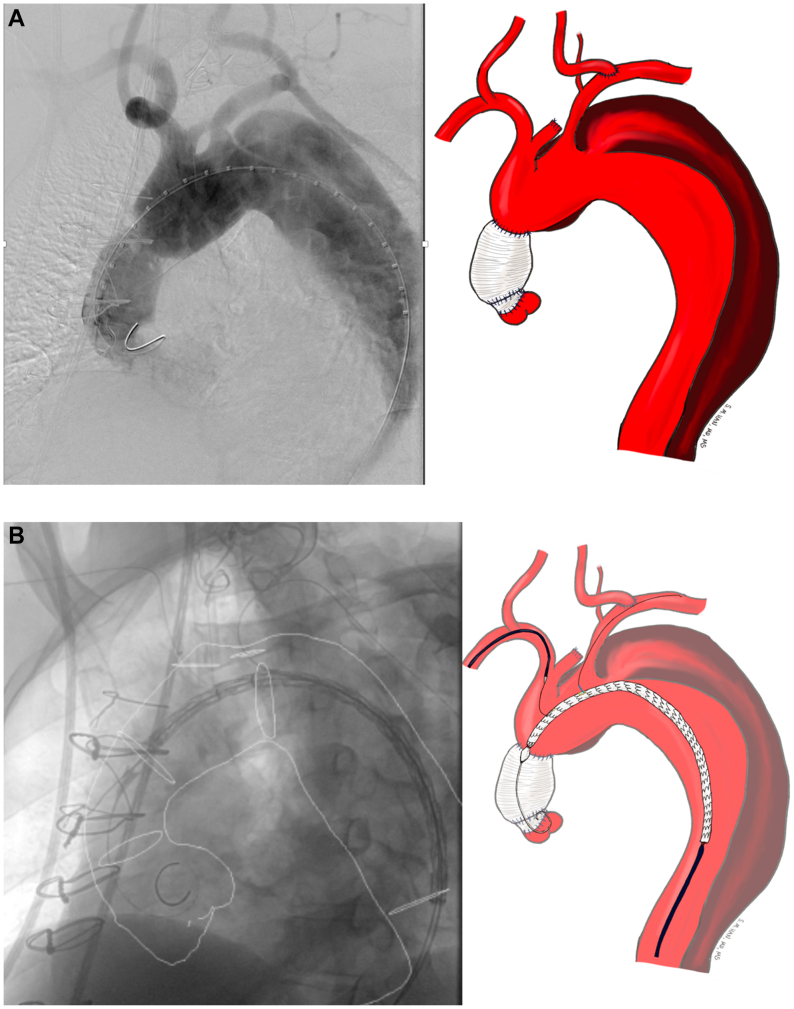

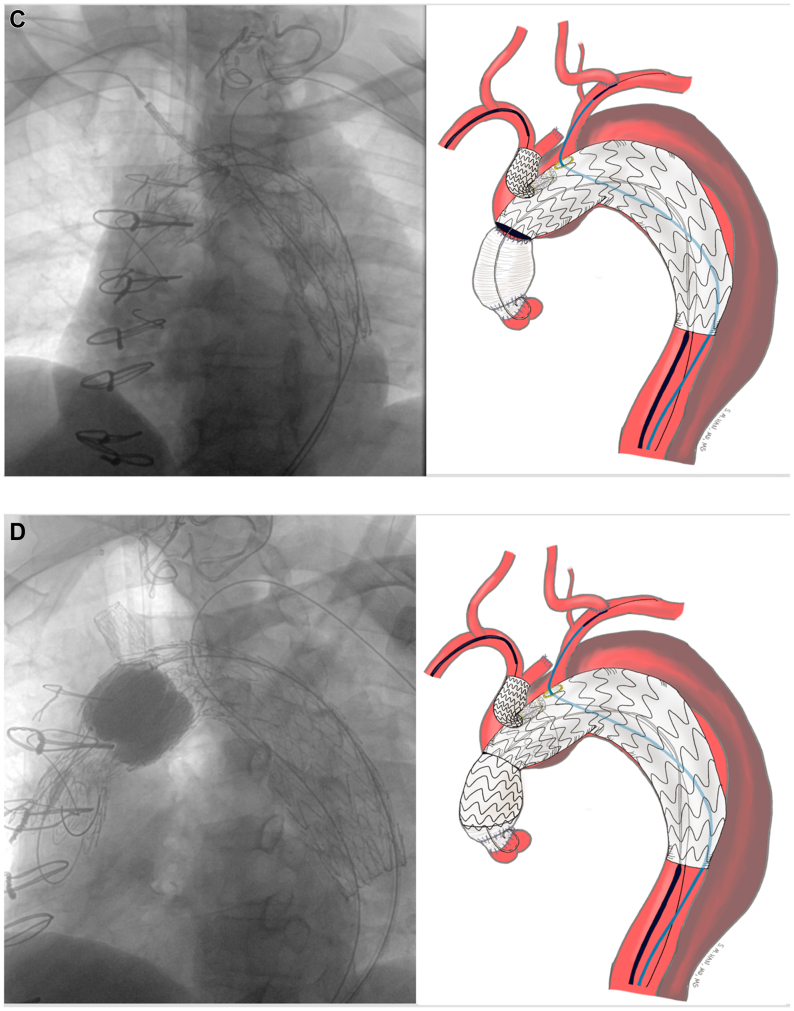

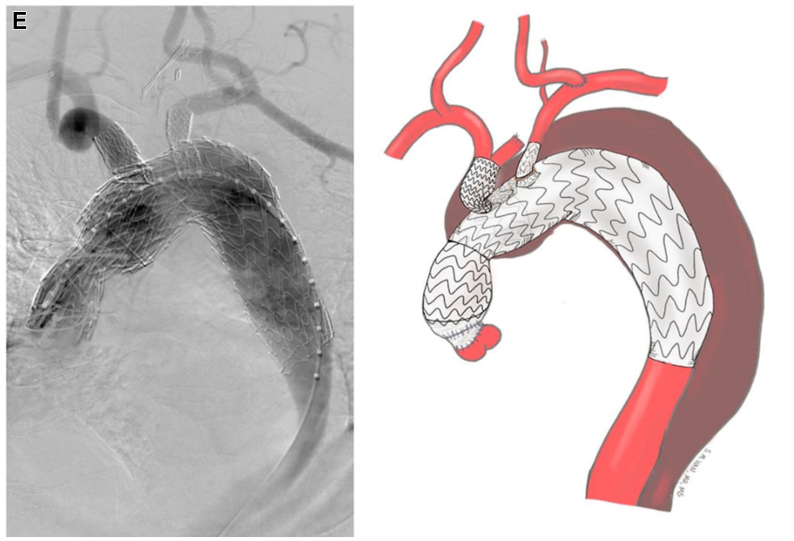
An additional fenestration within the existing Gore thoracic branch endoprosthesis platform (FEN-TBE) implantation steps. **(A)** Aortic arch after left carotid to subclavian transposition. **(B)** Undeployed FEN-TBE aligned with pre-wire in innominate and left subclavian cannulated with glidewire and 4Fr JR4 catheter. **(C)** Deployed FEN-TBE and right innominate stenting performed with right radial assistance. **(D)** Proximal extension deployed into ascending aorta. **(E)** Completed FEN-TBE with balloon expandable stent in the left subclavian artery (LSA) and Gore side branch in the innominate artery.

The preloaded FEN-TBE device is then advanced to align the portal to the innominate branch, while avoiding the wire wrap. While the device remains constrained and undeployed, the LSA is catheterized using the preloaded JR4 catheter and Glidewire (Fig 3). The brachial artery is selected, and an Amplatz wire is left in place for support with the preloaded catheter. Permissive hypotension is initiated in the 80 to 90 mmHg systolic range. We found rapid pacing or crossing the aortic valve unnecessary. The FEN-TBE device is then fully deployed. The TBE side branch component is delivered across the portal into the innominate artery over the through-and-through wire. Balloon molding of the seal zones are performed as needed, and the appropriately sized covered balloon expandible stents are placed across the LSA fenestration and flared (Fig 3).

## Results

A total of six patients underwent the Gore Fen TBE (Table). Of the cohort, five patients had undergone previous sternotomy in the past and were deemed high risk for revision open surgery. One patient was deemed too high risk for open surgery given his depressed ejection fraction. The in-hospital mortality and stroke rates were 0%. There were no chyle leaks associated with transposition. Patients were discharged on average by postoperative day 7. Periprocedural complications include one phrenic nerve injury, which recovered after 30 days. One patient required local debridement of a superficial infection of a percutaneous access site. Four patients were discharged home, and two were discharged to acute inpatient rehabilitation. All patients were home within 1 month of surgery. There were no endoleaks on postoperative imaging.

**Table:** Additional fenestration within the existing Gore thoracic branch endoprosthesis platform (FEN-TBE) case series including demographics, comorbidities, peri-procedural factors, devices utilized, and length of stay

Initials	Age, *years*	Gender	Race	Previous type A repair	CHF	Ejection fraction	Staged procedure	Arm access	Periprocedural complications	Stroke	Discharge postoperative day	Disposition	Endoleak	Innominate stent	LSA stent	Other medical comorbidities	Misc
TS	62	Male	African American	No	Yes	25%	No	Yes	None	None	8	Home	None	20 mm Gore Side Branch	11 × 39 VBX	HTN, COPD, tobacco use, Previous alcohol and cocaine use, Afib, NICM	Readmitted for COPD exacerbation but discharged home
RV	61	Female	African American	Yes	No	60%	No	Yes	None	None	12	Acute inpatient rehab	None	20 mm Gore Side Branch	11 × 39 VBX	HTN, HL, obesity, CKD- recent type A repair 3 months prior with rapid degeneration needing temporary dialysis, tracheostomy and PEG	Needed debridement of right groin for localized infection of percutaneous access site
DS	53	Male	African American	Yes	No	55%	Yes	Yes	None	None	11	Acute inpatient rehab	None	20 mm Gore Side Branch	11 × 59 VBX	HTN, HL, super obesity, previous type A repair and redo sternotomy with mechanical valve	
JO	68	Male	Hispanic	Yes	No	65%	No	Yes	None	None	4	Home	None	20 mm Gore Side Branch	11 × 39 VBX	HTN, previous type A repair	
SS	82	Male	White	Yes	No	63%	No	Yes	None	None	4	Home	None	20 mm Gore Side Branch	10 × 38 iCAST	HTN, HL, M, Afib, previous type A repair, chronic T-spine compression fractures	
AH	73	Male	African American	Yes	No	69%	No	Yes	None	None	3	Home	None	20 mm Gore Side Branch	11 × 39 VBX, 10 × 38 iCAST	HTN, PAF, DVT s/p IVC filter	

*Afib,* Atrial fibrillation; *CHF,* congestive heart failure; *CKD,* chronic kidney disease; *COPD,* chronic obstructive pulmonary disease; *DVT,* deep vein thrombosis; *HL,* hyperlipidemia; *HTN,* hypertension; *IVC,* inferior vena cava; *LSA,* left subclavian artery; *NICM,* nonischemic cardiomyopathy; *PAF,* paroxysmal atrial fibrillation; *PEG,* percutaneous endoscopic gastrostomy.

## Discussion

There are several advantages of the two-vessel FEN-TBE configuration compared with the single arch branch or multibranch constructs. Compared with the single branch zone 0 endovascular repair, this configuration reduces the complexity of complete cervical debranching to a single left carotid transposition. By limiting this to a single debranching procedure, there is reduced likelihood of local complications (stroke, chyle leak, phrenic nerve injury, dysphagia, globus sensation).^11^, ^12^, ^13^, ^14^, ^15^, ^16^ In addition, there is significantly less risk for infection, as the carotid transposition onto the LSA obviates the need for prosthetic graft. The carotid transposition onto the LSA, albeit unconventional, provides an autogenous configuration that facilitates an increased distance for accommodation of both a larger-diameter branch and a fenestration. Compared with the triple branch or fenestrated solution, the rotational forgiveness of the TBE inner branch with the ease of LSA catheterization simplifies the case planning to a single measurement of the innominate to LSA distance along the outer curve.^17^^,^^18^

There is limited ability to create fenestrations utilizing arc lengths without compromising device trackability and profile. However, with the pre-catheterized FEN-TBE approach, the device alignment in the arch is based on the LSA fenestration, and the innominate portal provides flexibility and forgiveness for the location of the innominate. It also offers protection against branch vessel instability and has wide applicability, as it includes patients with standard or bovine arch configurations. With the descending thoracic aorta considered to be a more fixed structure at the LSA, fenestrations may provide adequate stability, if there is not significant gap distance. As this modification does not introduce any structural alterations to the TBE stent, we do not anticipate additional device integrity issues such as stent fracture.

The transradial assisted access through the portal does not differ from the IFU zone 0 TBE implantation steps. Advantages inherent to the zone 0 TBE—namely excellent trackability and delivery to its intended location, alignment along the outer curve, and minimal ischemia time for the innominate—are entirely preserved with FEN-TBE. The TBE with radial-femoral access allows for excellent trackability and controlled alignment in the arch. This facilitates placement of the preloaded catheter along the outer curve and simplifies cannulation of the LSA prior to the device deployment. The device remains fully constrained with maximum space and forgiveness for cannulation of the LSA with no cerebral ischemic time.^19^ The short olive-tip eliminates the need to cross the aortic valve, as well as other hemodynamic adjuncts, such as rapid ventricular pacing. In our experience, transfemoral tracking of the available balloon expandable platforms with adequate stiff body wire through a fenestration has been excellent. Our approach mitigates the need for additional through wire left upper extremity access, thereby avoiding wire wrapping, which can be a significant issue and source of complication when multiple through and through access wires are utilized.

As with any endovascular arch repair involving cervical debranching, the decision to stage or not is nuanced and individually based on multiple factors: patient comorbidity, anatomic challenges of cervical debranching, and surgeon preference, to name a few. This decision-making is not unique to FEN-TBE. The majority of our patients were single-staged, as procedural times for the combined approach were all below 5 hours of total time under anesthesia.

## Conclusions

Our initial experience with FEN-TBE technique is encouraging and suggests that it provides a simplified hybrid strategy for endovascular arch repair with zone 0 proximal sealing. This approach is characterized by rapid modification, streamlined implantation, and unilateral cervical debranching. Further studies are planned to assess its long-term durability and learning curve associated with wider adoption.

## Funding

None.

## Disclosures

None.
